# Calpain inhibitor nanocrystals prepared using Nano Spray Dryer B-90

**DOI:** 10.1186/1556-276X-7-436

**Published:** 2012-08-04

**Authors:** Koichi Baba, Kohji Nishida

**Affiliations:** 1 Department of Ophthalmology, Osaka University Graduate School of Medicine, 2-2 Yamadaoka, Suita, Osaka, 565-0871, Japan

**Keywords:** Calpain inhibitor, Drug nanocrystals, Nano Spray Dryer B-90, 81.07.Wx, 81.16.-c

## Abstract

The Nano Spray Dryer B-90 offers a new, simple, and alternative approach for the production of drug nanocrystals. Among attractive drugs, calpain inhibitor that inhibits programmed cell death ‘apoptosis’ is a candidate for curing apoptosis-mediated intractable diseases such as Alzheimer’s disease and Parkinson’s disease. In this study, the preparation of calpain inhibitor nanocrystals using Nano Spray Dryer B-90 was demonstrated. The particle sizes were controlled by means of selecting mesh aperture sizes. The obtained average particle sizes were in the range of around 300 nm to submicron meter.

## Background

Using drug nanoparticles for a drug delivery system has attracted considerable attentions in the field of nanomedicine. Especially, nanoparticles with a drug filling rate of 100% including their amorphous form are called drug nanocrystals [1]. Since a nanocrystal has large surface area compared to a microparticle, the drug nanocrystal has several unique peculiarities such as its increased dissolution velocity, increased saturation velocity, and increased adhesiveness to cell membranes [2]. Additionally, drug nanocrystals are able to deliver large amount of drugs into cells and tissues at a single particle level because of their densely packed crystal structure [2]. Because of their unique physicochemical properties, recently, drug nanocrystals have been considered as a novel type of drug formulation for the drug delivery system [3].

Several approaches for preparing drug nanocrystals are classified as top-down and bottom-up procedures [4]. For the top-down procedures, techniques of milling, homogenization, and laser ablation are reported. For the bottom-up procedures, techniques of precipitation, chemical vapor, emulsion, and spray dryer are representatives. However, for the top-down procedures, all media milling processes involve high-energy input and are highly inefficient, and a considerable amount of heat is generated in these operations, making processing of thermolabile materials difficult. The contamination of milling balls that should be removed from drug dispersions by some efforts is also a problem, whereas for the bottom-up procedures, the removal of harsh solvents used for the preparation processes of drug dispersions is necessary in the precipitation technique [1]. Since the spray dryer technique takes a facile approach for preparing nanoparticles, which carries out spraying, evaporating the solvent-dissolved drug, and collecting drug particles, numerous drugs can be candidate for nanoparticle preparation in this technique. However, it is difficult for conventional spray dryer techniques to prepare particles with less than 2 μm in size and also to collect their fine particles [5]. In other words, submicrometer-sized particles, i.e., nanoparticles, cannot be obtained using the conventional spray dryer. Recently, an advanced spray dryer technology, Nano Spray Dryer B-90, has been developed by Büchi® (Flawil, Switzerland) [6]. The functions of piezoelectrically driven vibrating mesh and electrostatic particle collector realize the successful preparation and collection of nanoparticles. The different mesh aperture sizes create different sizes of nanoparticles. Currently, drug-encapsulated polymeric nanoparticles [7], protein nanoparticles [8], and lithium carbonate (Li_2_CO_3_) hollow spheres used in lithium batteries [9] have been successfully prepared using Nano Spray Dryer B-90.

**Figure 1:**
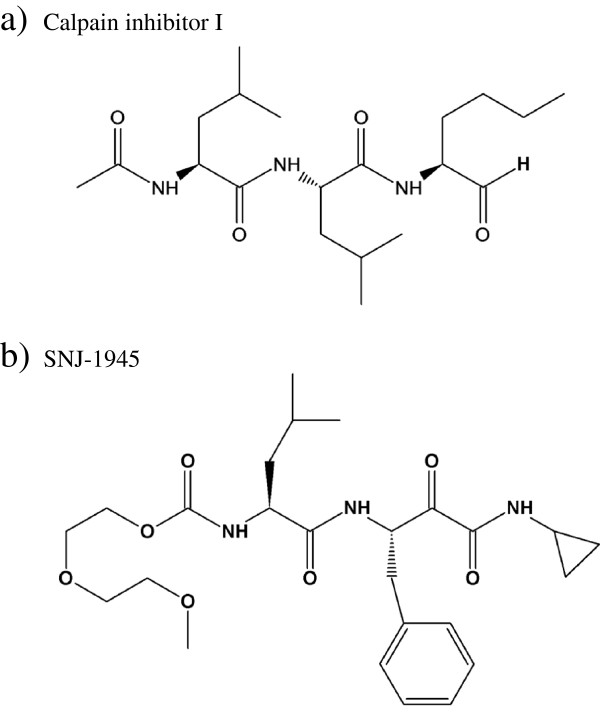
Chemical structures of calpain inhibitor I (a) and SNJ-1945 (b).

Recently, our interest is focused on preparing calpain inhibitor nanocrystals. Calpain inhibitors inhibit the calpain activity, resulting in reducing the calpain-meditated apoptosis [10]. Several kinds of calpain inhibitors have been synthesized such as leupeptin which improves motor neuron survival in rat embryos and calpain inhibitor VI (SJA6017) and calpain inhibitor XI [Z-L-Abu-CONH(CH2)3-morpholine] which are used for protecting both retinal and cortical neurons, respectively, against ischemia-induced damage [10]. Furthermore, calpain inhibitors are currently one of the most attractive drugs for curing intractable diseases such as Alzheimer’s disease and Parkinson’s disease [10]. In this research, we demonstrated the preparation of calpain inhibitor nanocrystals in powder conditions using Nano Spray Dryer B-90. The calpain inhibitor drugs selected were calpain inhibitor I (Figure 1a) [11] and ((1 S)-1-((((1 S)-1-benzyl-3-cyclopropylamino-2,3-di-oxopropyl)amino)carbonyl)-3-methylbutyl)carbamic acid 5-methoxy-3-oxapentyl ester (SNJ-1945; Figure 1b) [12]. Since it is known that the mesh aperture size significantly controls the resulting particle size [13], we investigated the relationship between mesh aperture size and particle size of drugs. Additionally, we briefly noted the effects of the concentration of dissolved drug solution, inlet temperature, and gas flow rate on the resulting particle size, especially targeting SNJ-1945 nanocrystals. 

## Methods

**Figure 2:**
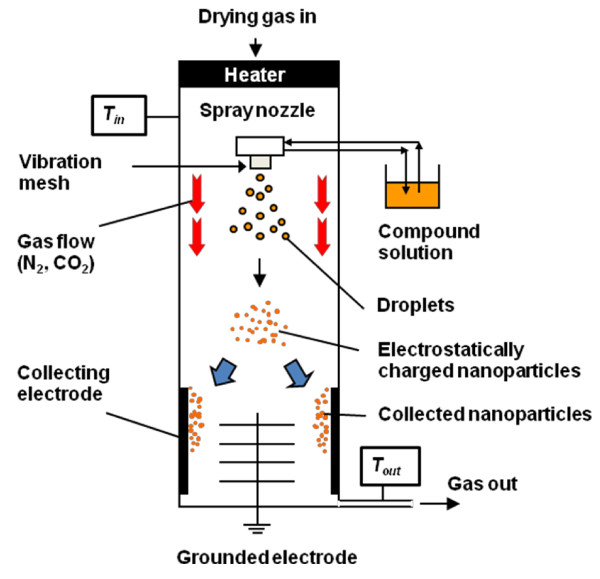
Schematic image of Nano Spray Dryer B-90.

The calpain inhibitor I and ethanol (99.5% *v*/*v*) were purchased from Wako Pure Chemical Industries (Osaka-shi, Osaka, Japan). The calpain inhibitor SNJ-1945 was kindly provided by Senju Pharmaceutical Co., Ltd. (Kobe-shi, Hyogo, Japan). Each drug was dissolved in ethanol (5 mg/10 ml). Then, each ethanol-dissolved drug solution was used for preparing each nanocrystal using Nano Spray Dryer B-90 (Büchi®). A schematic image of Nano Spray Dryer B-90 is illustrated in Figure 2. Briefly, the drying gas, which is heated up to the setting inlet temperature, flows into the drying chamber, and then the gas exits from the spray dryer passing through the clearing filter at the bottom. The inlet temperature and outlet temperature are shown as *T*_in_ and *T*_out_, respectively. The operating conditions for the experiments were kept constant at *T*_in_ = 50°C, *T*_out_ = 35°C, feed rates of 25 ml/h, and a drying gas flow rate of 100 L/min. Spray mesh aperture sizes of 4.0, 5.5, and 7.0 μm were used in this experiment. Finally, the resulting nanocrystal powders were collected using a rubber spatula. The morphology and size of collected particles were observed by scanning electron microscopy (SEM; JEOL-6510LA, Akishima-shi, Tokyo, Japan). The average size and particle size distribution were calculated by means of counting more than 300 particles from the obtained SEM images.

## Results and discussion

**Figure 3:**
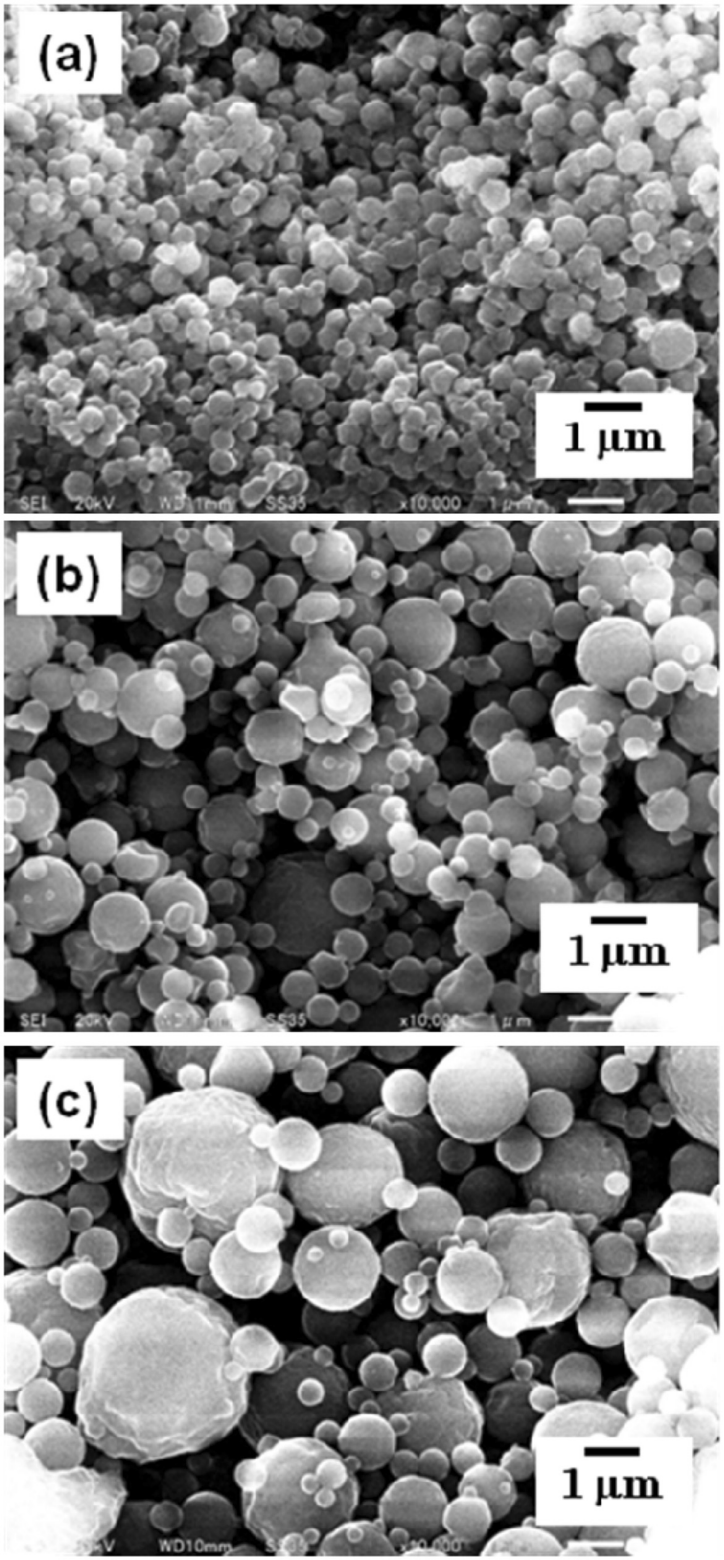
**SEM images of calpain inhibitor I nanocrystals.** The nanocrystals were prepared using mesh with aperture sizes of 4.0 (**a**), 5.5 (**b**), and 7.0 μm (**c**).

**Figure 4:**
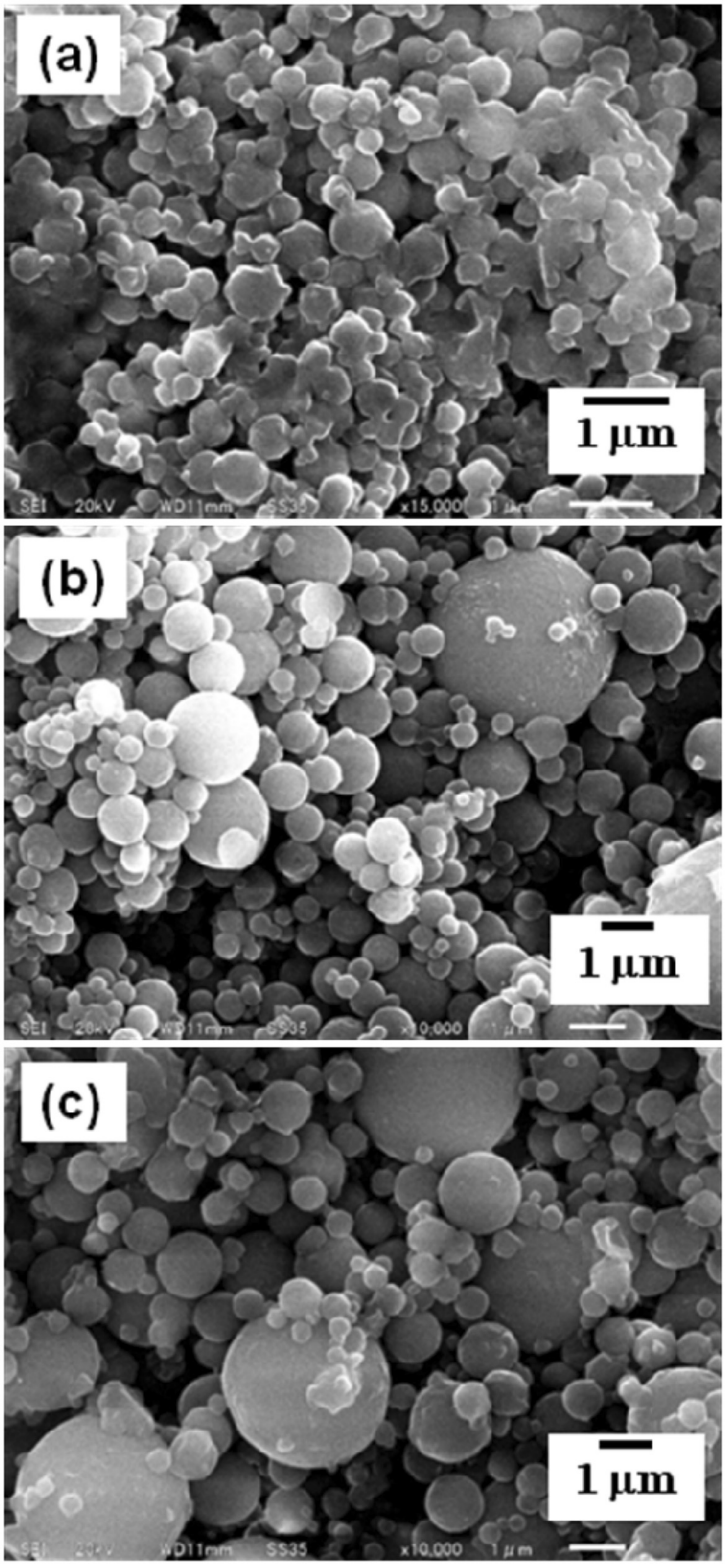
**SEM images of SNJ-1945 nanocrystals.** The nanocrystals were prepared using mesh with aperture sizes of 4.0 (**a)**, 5.5 (**b**), and 7.0 μm (**c**).

**Figure 5:**
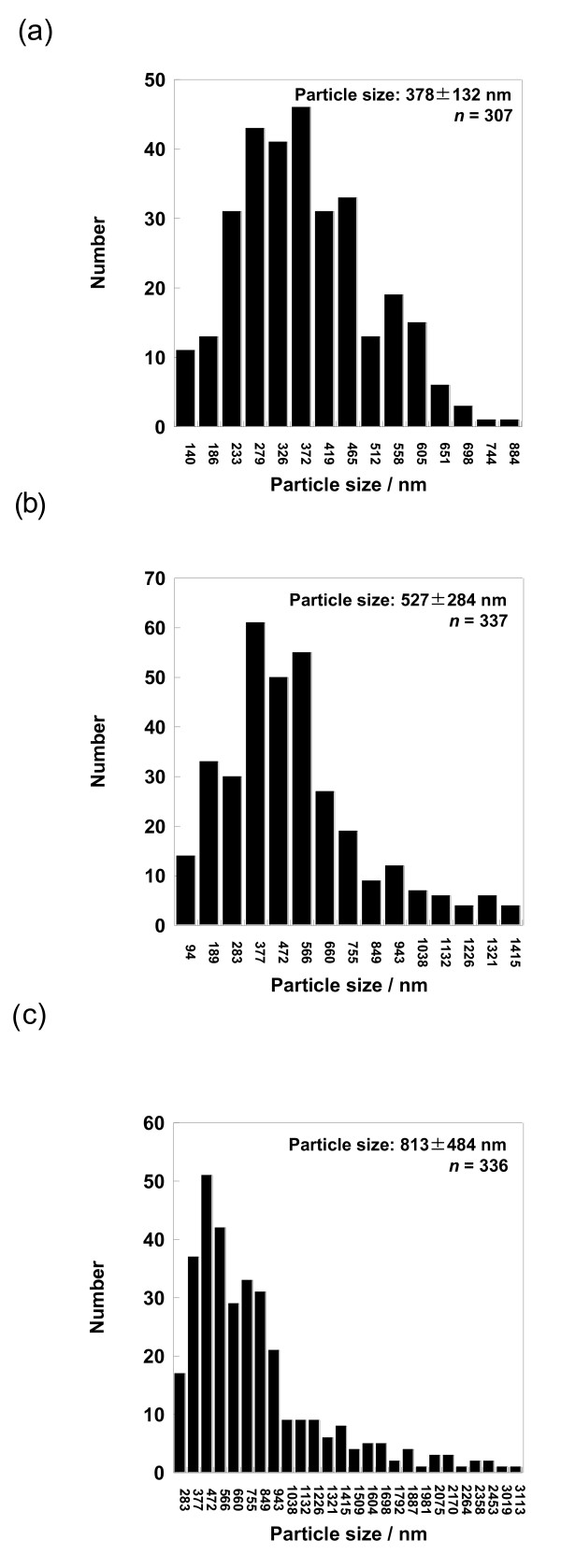
**Average sizes with their size distribution of calpain inhibitor I nanocrystals.** The used mesh aperture sizes were 4.0 (**a**), 5.5 (**b**), and 7.0 μm (**c**). ‘*n*’ represents the number of counted particles.

**Figure 6:**
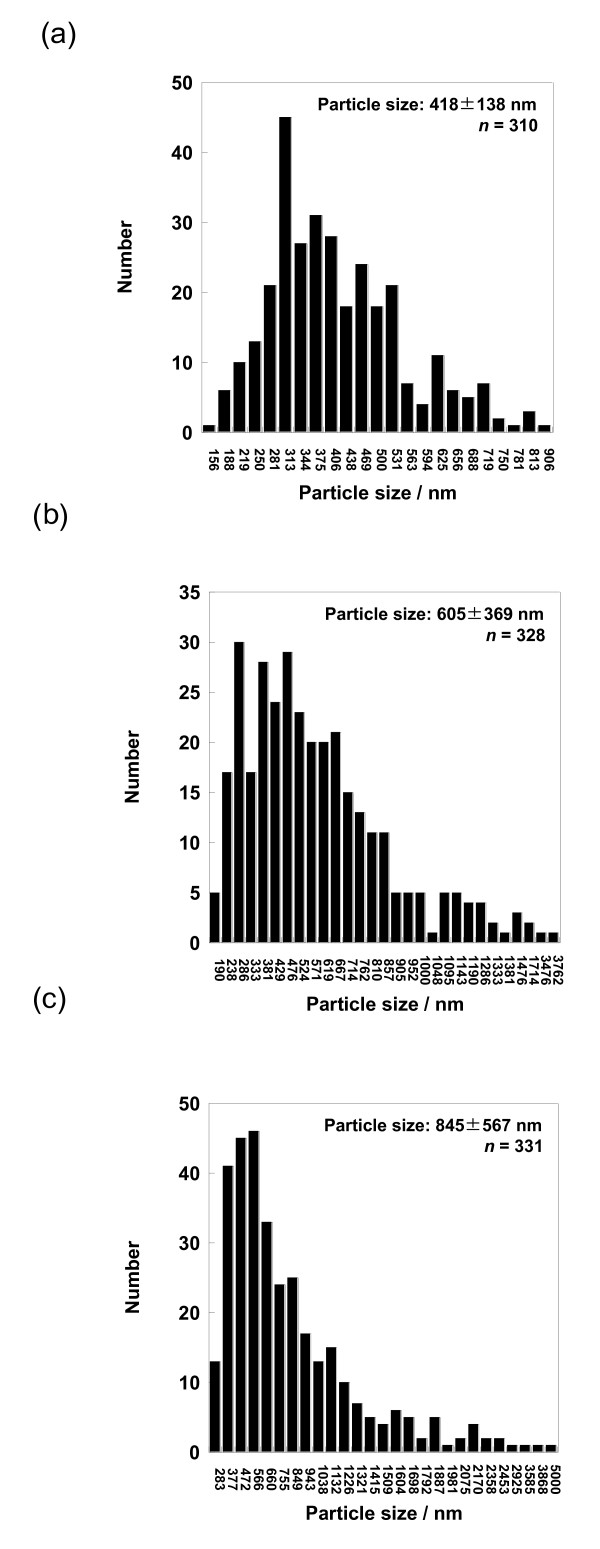
**Average sizes with their size distribution of SNJ-1945 nanocrystals.** The used mesh aperture sizes were 4.0 (**a**), 5.5 (**b**), and 7.0 μm (**c**). ‘*n*’ represents the number of counted particles.

The ethanol-dissolved drug solution was fed to the spray head by pumping. The solution was atomized by piezoelectrically driven mesh vibration in a small spray cap, and then millions of precisely sized droplets per second with a narrow distribution were ejected by the vibrating actuator driven at around 60 kHz. The extremely fine droplets were dried to become solid particles while passing through the chamber, and then these particles, which were electrostatically charged by dry gases of N_2_ and CO_2_, were collected by the electrode. The collected particles using the rubber spatula were observed by SEM. The morphology of each calpain inhibitor nanocrystal was sphere-like regardless of mesh aperture sizes of 4.0, 5.5, and 7.0 μm (Figures 3 and 4). On the other hand, their particle sizes were associated with the mesh aperture sizes. For the calpain inhibitor I nanocrystals, the average particle sizes with their size distribution were 378 ± 132, 527 ± 284, and 813 ± 484 nm against mesh aperture sizes of 4.0, 5.5, and 7.0 μm, respectively (Figure 5). For the SNJ-1945 nanocrystals, the average particle sizes with their size distribution were 418 ± 138, 605 ± 369, and 845 ± 567 nm against mesh aperture sizes of 4.0, 5.5, and 7.0 μm, respectively (Figure 6). For both cases of calpain inhibitor I and SNJ-1945 nanocrystals, the average particle size as well as their size-distribution width relating to particle polydispersity became small and narrow with decreasing mesh aperture sizes, respectively. The validity of these results is supported by previous reports that the different mesh aperture sizes resulted in making different sizes with size-distribution widths of particles, e.g., average particle size and size-distribution width decreased with decreasing mesh aperture size [13]. This is because the small mesh aperture size (e.g., 4.0 μm) tends to generate small-sized droplets of the ethanol-dissolved drug solution, which results in creating small-sized particles with narrow size-distribution widths, compared to utilizing the large mesh aperture size (e.g., 7.0 μm). We revealed that the selection of mesh aperture sizes controlled the particle sizes of calpain inhibitor I and SNJ-1945 nanocrystals. 

**Figure 7:**
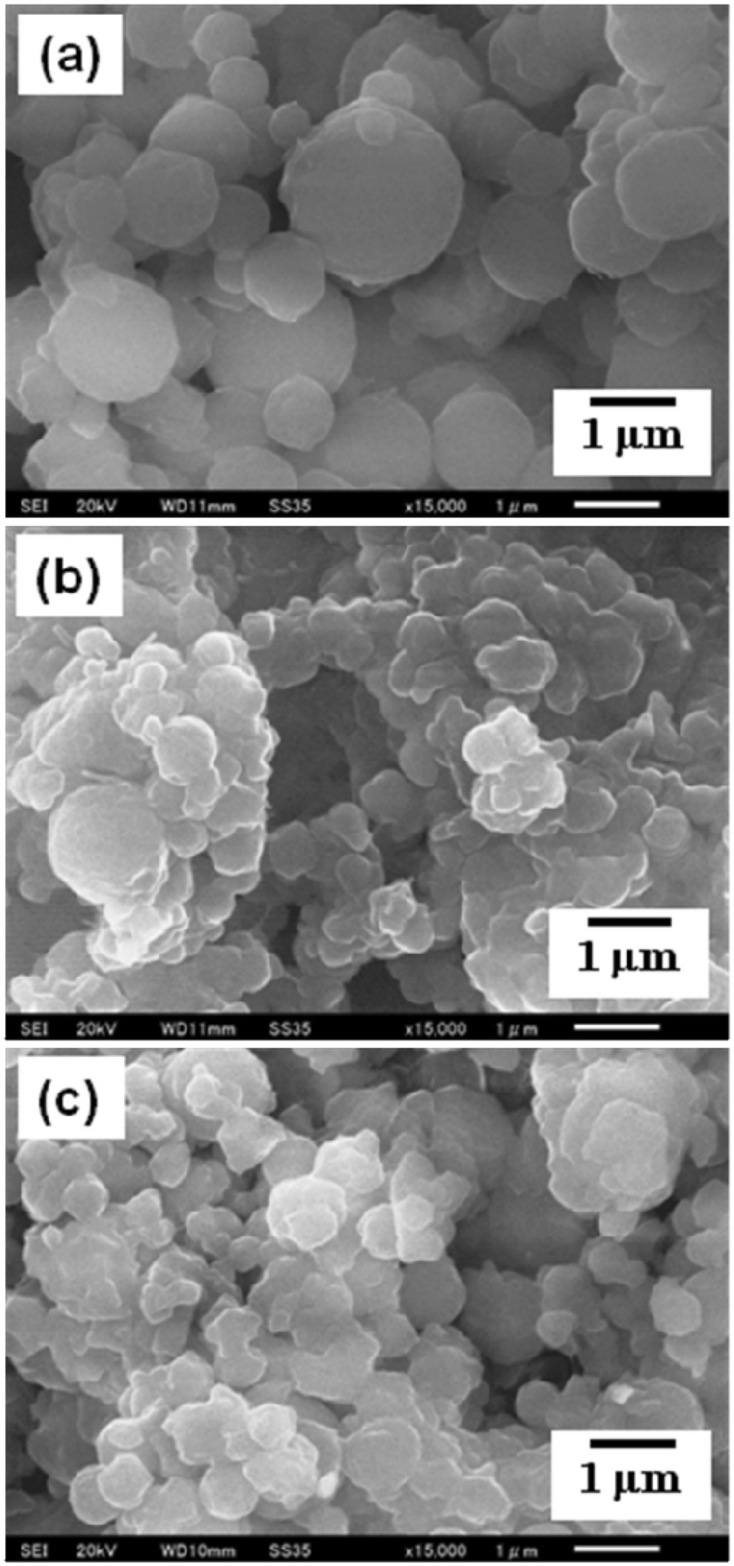
**SEM images of SNJ-1945 nanocrystals.** The nanocrystals were prepared using mesh with aperture size of 4.0 μm at different experimental conditions, especially focusing on concentration (**a**), *T*_in_ (**b**), and gas flow rate (**c**).

**Figure 8:**
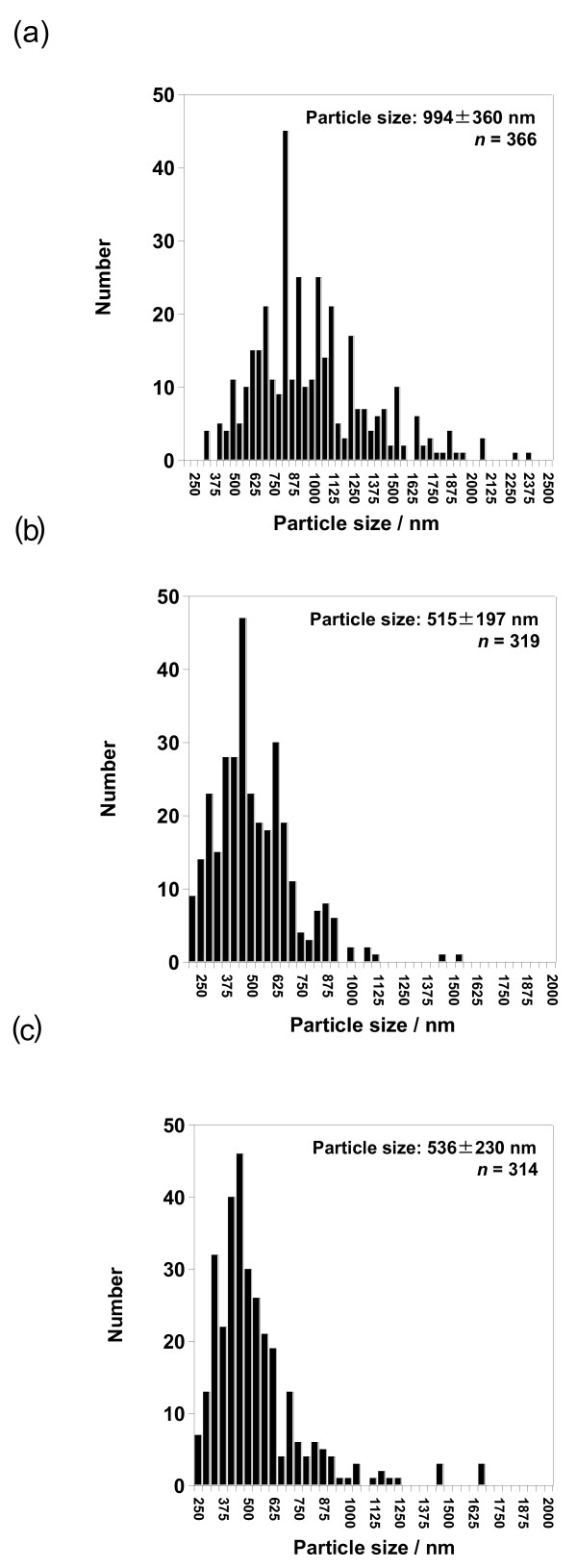
**Average sizes with their size distribution of SNJ-1945 nanocrystals.** The nanocrystals were prepared using mesh with aperture size of 4.0 μm at different experimental conditions, especially focusing on concentration (**a**), *T*_in_ (**b**), and gas flow rate (**c**). ‘*n*’ represents the number of counted particles.

We also investigated the effects of the experimental conditions relating to the concentration of ethanol-dissolved drug solution, *T*_in_, and gas flow rate on the resulting particle size, which were compared with those of the standard sample of SNJ-1945 nanocrystals. The standard sample of SNJ-1945 nanocrystals (418 ± 138 nm in size) was prepared with the following experimental conditions: concentration = 5 mg/10 ml, *T*_in_ = 50°C, gas flow rate = 100 L/min, and mesh aperture size = 4.0 μm (see the data of the standard sample in Figures 4a and 6a, and case I in Table 1). It was revealed that the concentration significantly affected the particle size, namely when the concentration increased tenfold (50 mg/10 ml), the particle size increased almost twice compared to the effect of utilizing the concentration (5 mg/10 ml) of the standard sample (cases I and II in Table 1). The image of the particles obtained by SEM observation is shown in Figure 7a. The average particle size and their size distribution was 994 ± 360 nm (Figure 8a). On the other hand, the conditions of high *T*_in_ (100°C) and high gas flow rate (150 L/min) did not significantly affect the particle size compared to the effect of utilizing high concentration of 50 mg/10 ml (cases I to IV in Table 1). The images of the particles obtained by SEM observation at the conditions of *T*_in_ = 100°C and gas flow rate = 150 L/min are shown in Figure 7b,c, respectively. The average particle size and their size distribution at the conditions of *T*_in_ = 100°C and gas flow rate = 150 L/min were 515 ± 197 and 536 ± 230 nm, respectively (Figure 8b,c). These obtained results seemed to be reasonable when considering previous reports [13,14], namely the particle size was significantly affected by the concentration of ethanol-dissolved drug solution, but not significantly by *T*_in_ and gas flow rate. More detailed experimental conditions related to the concentration of dissolved drug solution, temperature, and gas flow rate, which will affect particle formation, will be investigated as future works including several kinds of calpain inhibitor nanocrystals. 

**Table 1:** Particle size and size distribution of SNJ-1945 nanocrystals prepared at different experimental conditions

**Experimental conditions**	**Samples**			
	**Case I**	**Case II**	**Case III**	**Case IV**
Concentration (mg/ml)	5:10	50:10	5:10	5:10
*T*_in_ (°C)	50	50	100	50
Flow rate (L/min)	100	100	100	150
Particle size and size distribution (nm)	418 ± 138	994 ± 360	515 ± 197	536 ± 230

The mesh aperture size used was 4.0 μm.

## Conclusions

We succeeded in preparing size-controlled calpain inhibitor nanocrystals using Nano Spray Dryer B-90. To the best of our knowledge, this is the first time to prepare calpain inhibitor nanocrystals. The particle sizes were associated with the mesh aperture sizes, namely when the mesh aperture sizes decreased, the particle sizes decreased. The concentration of ethanol-dissolved drug solution significantly affected the particle size. Calpain inhibitors will be a promising drug for curing intractable diseases such as Alzheimer’s disease and Parkinson’s disease. Therefore, utilizing the calpain-inhibitor-nanocrystal-based drug delivery system targeting such intractable diseases will be quite attractive topics in the near future, and thus our fundamental findings of preparing calpain inhibitor nanocrystals in this research are noteworthy. We are also investigating the preparation of water dispersion of calpain inhibitor nanocrystals. This nanocrystal water dispersion will act as attractive nanocrystal-based eye drops for the treatment of ophthalmic disorders such as Fuchs’ endothelial dystrophy of the cornea in the near future.

## Competing interests

The authors declare that they have no competing interests.

## Authors’ contributions

KB contributed to the conception of the study, carried out all the experiments, and drafted the manuscript. KN contributed to the interpretation of data and revised the manuscript. Both authors read and approved the final manuscript.

## Authors’ information

KB is a specially appointed associate professor and KN is a professor and a medical doctor at the Department of Ophthalmology, Osaka University Graduate School of Medicine, Japan.
